# SIRT3 Enhances Glycolysis and Proliferation in SIRT3-Expressing Gastric Cancer Cells

**DOI:** 10.1371/journal.pone.0129834

**Published:** 2015-06-29

**Authors:** Yang Cui, Lili Qin, Jing Wu, Xuan Qu, Chen Hou, Wenyan Sun, Shiyong Li, Andrew T. M. Vaughan, Jian Jian Li, Jiankang Liu

**Affiliations:** 1 Center for Mitochondrial Biology and Medicine, Key Laboratory of Biomedical Information Engineering of Ministry of Education, School of Life Science and Technology and Frontier Institute of Life Science, Xi’an Jiaotong University, Xi’an, 710049, China; 2 Department of Radiation Oncology, NCI-designated Compressive Cancer Center, University of California Davis, Sacramento, California, 95817, United States of America; 3 Department of Clinical Pathology, Emory University, Atlanta, Georgia, 30322, United States of America; University of Kentucky, UNITED STATES

## Abstract

SIRT3 is a key NAD^+^-dependent protein deacetylase in the mitochondria of mammalian cells, functioning to prevent cell aging and transformation via regulation of mitochondrial metabolic homeostasis. However, SIRT3 is also found to express in some human tumors; its role in these SIRT3-expressing tumor cells needs to be elucidated. This study demonstrated that the expression of SIRT3 was elevated in a group of gastric cancer cells compared to normal gastric epithelial cells. Although SIRT3 expression levels were increased in the gastric tumor tissues compared to the adjacent non-tumor tissues, SIRT3 positive cancer cells were more frequently detected in the intestinal type gastric cancers than the diffuse type gastric cancers, indicating that SIRT3 is linked with subtypes of gastric cancer. Overexpression of SIRT3 promoted cell proliferation and enhanced ATP generation, glucose uptake, glycogen formation, MnSOD activity and lactate production, which were inhibited by SIRT3 knockdown, indicating that SIRT3 plays a role in reprogramming the bioenergetics in gastric tumor cells. Further analysis revealed that SIRT3 interacted with and deacetylated the lactate dehydrogenase A (LDHA), a key protein in regulating anaerobic glycolysis, enhancing LDHA activity. In consistence, a cluster of glycolysis-associated genes was upregulated in the SIRT3-overexpressing gastric tumor cells. Thus, in addition to the well-documented SIRT3-mediated mitochondrial homeostasis in normal cells, SIRT3 may enhance glycolysis and cell proliferation in SIRT3-expressing cancer cells.

## Introduction

Sirtuins, a family of NAD^+^-dependent histone deacetylases (HDACs) in mammalian cells, are implicated in a wide range of physical processes including cell survival, apoptosis, metabolism, stress responses, aging and longevity [1,2]. Among seven sirtuin members (SIRT1–7), SIRT3 is the best characterized mitochondrial sirtuin, functioning to regulate mitochondrial proteins involved in oxidative phosphorylation, fatty acid oxidation, the urea cycle, and the antioxidant response [2–9]. Several studies have highlighted the role of SIRT3 in metabolism and homeostasis in normal cells and revealed new targets and substrates for SIRT3-dependent deacetylation [10]. Kim et al reported that SIRT3 is a key mitochondria protein, and lack of the SIRT3 expression is linked to increased mitochondrial DNA damage and aging, as well as increased potential to Ras-induced cell transformation and SIRT3-mediated MnSOD activation contributing to the mitochondrial homeostasis [11,12]. In support, human embryonic kidney 293 cells (HEK293) cells exhibit an enhanced SIRT3 expression under oxidative stress, leading to deacetylation and activation of MnSOD [13]. SIRT3 is believed to function as a tumor suppressor gene and plays a key role in enhancing cell homeostasis against aging and carcinogenesis.

However, some tumor cells show the expression of SIRT3 and the potential role of SIRT3 in these tumor cells, especially its potential relation to the aggressive phenotype, has been controversial [14]. SIRT3 expression is lower or undetectable in an array of human cancers, including breast cancer, glioblastoma, colon cancer, and osteosarcoma, prostate and ovarian cancers [11,15,16]. SIRT3 induces growth arrest and apoptosis by selective silencing of Bcl-2 in HCT116 cells through modulating JNK2 signaling pathway [17]. Also, SIRT3 is reported to contribute to increased sensitivity of human leukemia cells to chemotherapy possibly through the induction of mitochondria-mediated apoptosis [18]. On the other hand, SIRT3 expression is also found be increased in oral cancer, node-positive breast cancer, esophageal cancer, and thyroid carcinomas; and the increased SIRT3 is associated with higher malignant phenotype and downregulation of SIRT3 enhances tumor sensitivity to anti-cancer treatment [19–23]. These results alert a different role of SIRT3 in specific tumors that needs to be elucidated.

Cancer cells are metabolically active and consume more cellular fuel than normal cells. However cancer cells relay on mainly on the ATP synthesis by aerobic glycolysis, a feature known as Warburg effect [24]. Such an aerobic glycolysis is believed to protect cancer cells form oxidative stress since mitochondrial respiration is the main source of intracellular ROS [25]. The lactate dehydrogenase A (LDHA) is an enzyme controlling interconversion between pyruvate and L-lactate reversibly at the final step of the anaerobic glycolysis [26]. Inhibition of LDHA diminishes the tumorigenic potential with increased mitochondrial oxygen consumption and decreased mitochondrial membrane potential [26] and overall cellular ATP production and glycolysis [27].

In this study, we investigated the potential role of SIRT3 in gastric cancer cells that express SIRT3. Expression of SIRT3 was linked with the aggressive growth, which was mediated via SIRT3-deacetylated and activated LDHA, enhancing glycolysis and expression of a group of glycolysis-associated genes. Thus SIRT3-LDHA-mediated glycolysis metabolism may be a potential therapeutic target to treat cancer cells that express SIRT3.

## Materials and Methods

### Cell lines and culture

Human gastric cancer cell lines MGC-803, HGC-27, SGC-7901 [28] and AGS [29] and immortalized human gastric epithelial cell line GES-1 [30] were maintained in RPMI-1640 medium (Invitrogen) supplemented with 10% fetal bovine serum and 1% penicillin/streptomycin, and cultured at 37°C under an atmosphere of 5% CO2.

### Immunoprecipitation and western blot

Subconfluent cells were lysed and the lysates were incubated with protein A/G agarose beads plus the recommended amount of antibodies against either SIRT3 (Cell Signaling Technology) or LDHA (Santa Cruze) at 4°C overnight. Samples were centrifuged and separated by SDS polyacrylamide gel electrophoresis and electroblotted onto nitrocellulose membranes. After blocking with 5% non-fat milk in TBST, membranes were incubated with primary antibody at 4°C overnight followed by incubation with secondary antibody for 1 hour at RT. Proteins of interest were visualized by ECL detection kit (Thermo Fisher).

### Immunohistochemistry assay

Pathological slides of gastric cancer with adjacent normal tissues were analyzed by immunohistochemistry assay were performed following the manufacturer’s instructions. The Polymer Detection kit for the immunohistochemistry analysis was purchased from Zhongshan Golden Bridge Biotechnology. Briefly, paraffin sections were dewaxed and re-hydrated in ethanol (100%, 90% and 75%). The sections were incubated with 3% H_2_O_2_ at RT for 10 minutes and then blocked with non-immune goat serum at RT for 15 minutes. The sections were then incubated with primary antibody to SIRT3 (Cell Signaling Technology) for 2 hours at RT followed by anti-rabbit secondary antibody incubation for 15 minutes at RT. The sections were then incubated with DAB detection reagent for 2 minutes each, counterstained with hematoxylin and dehydraded in ethanol (90% and 100%). The sections were mounted and the staining was analyzed under the microscopy.

### Plasmid construction and cell transfection

SIRT3 whole length cDNA was synthesized using RT-PCR with total RNA extracted from GES-1 cells as template (primers: 5’ CCGCGGTACCATGGCGTTGTGGGGTTG 3’ and 5’ CCGCTCTAGACTATTTGTCTGGTCCATCAAGC 3’). The cDNA was then cloned into pcDNA3.1+ expression vector (Invitrogen). The pGPH1/GFP/Neo-SIRT3-shRNA plasmid targeting human SIRT3 (5’ CTTGCTGCATGTGGTTGAT 3’) and the control shRNA plasmid were obtained from GenePharma. To generate stable transfectants, AGS and SGC-7901 cells were transfected using Lipofectamine 2000 reagent (Invitrogen) and the stable transfectants were selected by 400ug/ml G418 (Gibco).

### Cell proliferation and clonogenicity assay

For proliferation assay, cells were plated in a 6-well plate at 2 x 10^4^ cells/well. Cell proliferation was calculated on days 2, 4, 6, and 8 after plating. For clonogenicity assay, cells were plated in a 6-well plate with varied cell numbers and cultured for 14 days and colonies were then stained with crystal violet, and the number of colonies (> 50 cells/colony) was counted following the established methods [31].

### Measurement of glucose, lactate and glycogen

The phenol red free medium was used to culture cells in 6-well plate for 24 hours and the levels of glucose uptake and lactate generation were measured using the Glucose Assay Kit and Lactic Acid Kit (Jiancheng Bioengineering Institute). The relative values were normalized to the cell number of each well. Cellular glycogen levels were detected using the Glycogen Assay Kit (Biovision). Briefly, 1×10^6^ cells were homogenized in 200μL water on ice, and the homogenates were boiled for 5 minutes to inactivate enzymes and centrifuged at 13,000 rpm for 5 minutes at 4°C to remove insoluble material. The glycogen production was measured with the OD value at 570 nm.

### Measurement of ATP production

Cellular ATP and ROS levels were measured as described previously [32]. Cells cultured in a 6-well plate after various treatments, were lysed and cellular ATP levels were measured with ATP Bioluminescence Assay Kit (Sigma). For measuring glycolysis-mediated ATP production, cells were treated with 2 μM rotenone for 24 hours before measurements.

### Measurement of ROS generation

Cellular ROS generation was assayed with 5-(and 6-) carboxy-2’,7’-dichlorodihydrofluorescein diacetate (DCFDA) method using microplate spectrometer (Thermo Fisher) at 488 nm/530 nm wavelength.

### MnSOD activity

Cells cultured in a 6-well plate were lysed and MnSOD activity was determined by WST-8 methods using the MnSOD assay kit (Beyotime) following the manufacturer’s instructions.

### Tumorigenicity assays in nude mice

SGC7901 cells (7.5 x 10^6^) with SIRT3 overexpression or knockdown were suspended in 0.1 ml PBS and inoculated into either flank of 5-week-old male BALB/c athymic nude mice, and at the 28^th^ day after inoculation when the maximal tumor volume (~1500 mm^3^) reached in the control group, the experiments were terminated and all of tumors from each group were excised and weighed. The experimental procedure involving animal tests was conducted in accordance with the institutional guidelines; the Institutional Animal Care and Use Committee (IACUC) at Xian Jiao Tong University specifically approved this study (Protocol # 120181).

### Lactate dehydrogenase assay

The lactate dehydrogenase activity was determined following the established protocol by measuring the reduction rate in the absorbance at wavelength of 340 nm resulting from the oxidation of NADH [27]. Briefly, cells were cultured in 10 cm dishes and homogenized in PBS on ice. The homogenates were added into buffer containing 6.6 mM NADH, 30 mM sodium pyruvate and 0.2 M Tris-HCl, pH 7.3 and mixed. Incubate the mixture for 5 minutes to achieve temperature equilibration and then record the decrease rate of absorbance at 340 nm.

### SIRT3 *in vitro* deacetylation assay

Human SIRT3 recombinant enzyme (BPS) was incubated with L-Lactic Dehydrogenase from bovine heart (Sigma) in reaction buffer (50 mM NaCl, 4 mM MgCl_2_, 10 mM NAD^+^, 50 mM DTT, 50 mM Tris, pH 8.0) with/without SIRT3 inhibitor nicotinamide (NAM, 10 mM) at 37°C for 1.5 hours, and then the reactions were used for LDHA activity assay described as above.

### Real-time PCR

Total RNA was isolated using TRIZOL Reagent (Invitrogen) followed by treatment with phenol/chloroform and precipitation with 2-propanol. Total RNA pellet was then washed with 75% ethanol and resuspended in water. RNA was subjected to reverse transcription with PrimeScript RT-PCR Kit (Takara) and quantitative real-time PCR analysis of genes of interest was conducted using SYBR PremixExTaq II kit (Takara) following the manufacturer’s instructions. Data were normalized to the level of γ-tubulin.

### Immunofluorescence

AGS cells seeded on the coverslip in a 6-well plate were fixed in 4% paraformaldehyde for 10 minutes and blocked in 1% BSA for 1 hour at RT. Incubate cells with primary antibodies against SIRT3 and LDHA at 4°C overnight, followed by incubation with fluorescence-conjugated secondary antibody for 1 hour at RT. Counterstaining was carried out with DAPI using immunofluorescence kit (Beyotime). Cells were mounted and imaged by laser scanning confocal microscope.

### Statistical analysis

Data are presented as mean ± SE from at least three independent experiments. Statistical analysis was performed with unpaired two-tailed Student’s t test unless otherwise noted. *P* < 0.05 was considered significant.

## Results

### SIRT3 expression in gastric cancer cells

**Fig 1:**
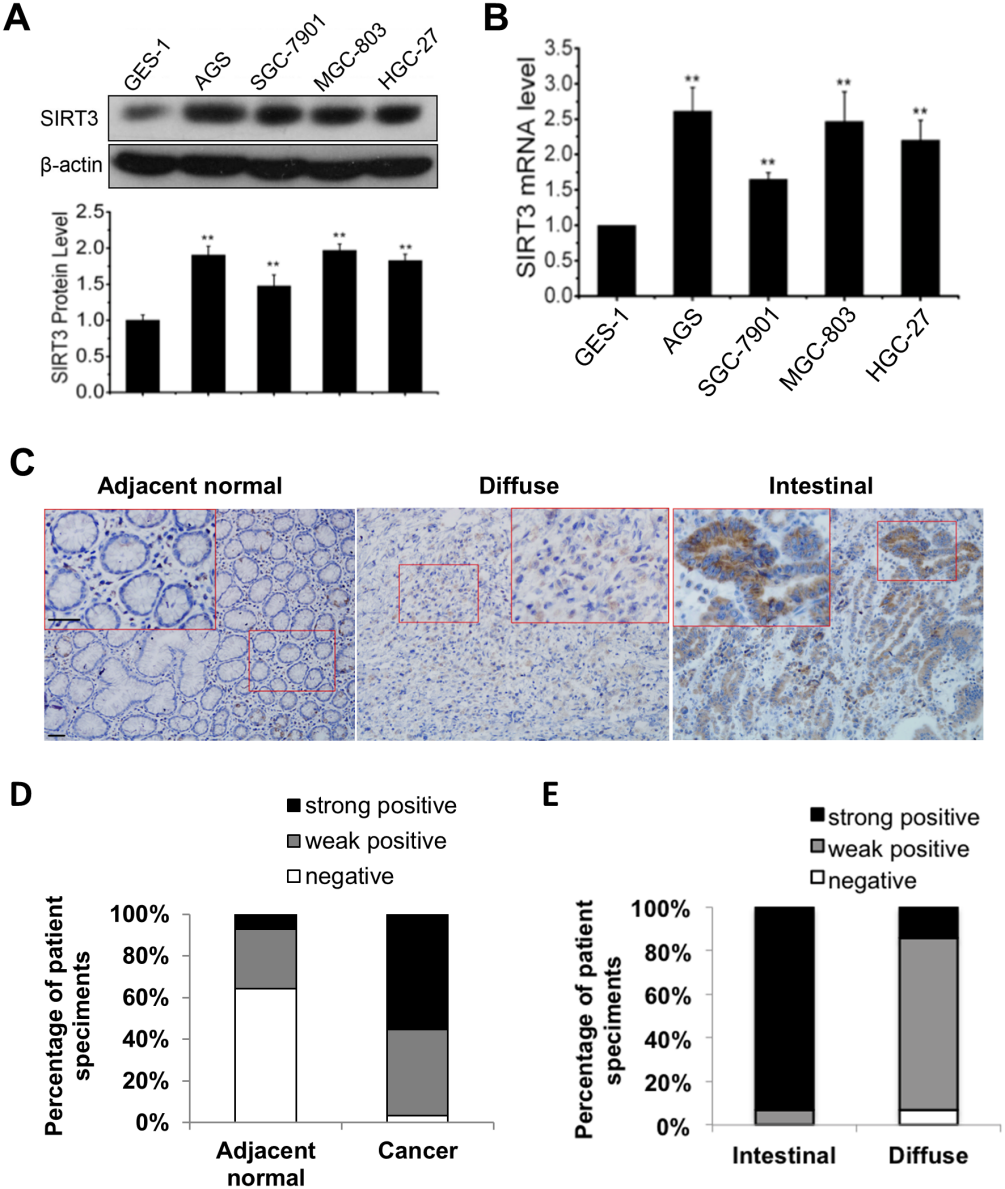
Enhanced SIRT3 expression in human gastric cancer cell lines and tumor tissues. A, SIRT3 protein levels were determined by western blot in 4 gastric cancer cell lines and an immortalized normal gastric epithelium cell line GES-1 (upper panel). SIRT3 protein expression tested in three separate western blots was estimated by measuring the band intensity using Image-Pro Plus software and normalized with β-actin (lower panel). B, SIRT3 mRNA levels were detected by qRT-PCR in 4 gastric cancer cell lines. Data were normalized to immortalized normal gastric epithelium cells. In (A, B), data are presented as mean ± S.E. (n = 3; *, *p* < 0.05; **, *p* < 0.01). C, SIRT3 expression in human gastric tumor tissues (n = 29) and adjacent non-tumor tissues (n = 14) was detected by immunohistochemistry staining. SIRT3 expression levels were estimated by density scanning using Image-Pro Plus software and graded as negative, weak positive and strong positive. Scale bar, 50 μm. D, SIRT3 expression in tumor and adjacent non-tumor tissues was presented as percentage of patient specimens. E, SIRT3 expression in intestinal (n = 18) and diffuse (n = 11) types of gastric tumor tissues was presented as percentage of patient specimens.

SIRT3 is well-defined in regulating mitochondrial homeostasis in anti-aging and anti-transformation. Recently, different results are reported regarding to SIRT3-mediated cell inhibition and proliferation, leading to the controversy of its roles in cancers [19,20]. To determine the potential role of SIRT3 in gastric cancer, expression of SIRT3 was detected in 4 gastric cancer cell lines AGS, SGC-7901, MGC-803, HGC-27 and a group of gastric cancer tumor tissues. We found that SIRT3 protein levels were elevated in all 4 gastric cancer cell lines compared to the immortalized gastric epithelial GES-1 cells (AGS, 1.9-fold; SGC-7901, 1.5-fold; MGC-803, 2.0-fold; and HGC-27, 1.8-fold compared to GES-1 cells) (Fig 1A). In accordance, the mRNA levels of SIRT3 in these cell lines were also increased (AGS, 2.6-fold; SGC-7901, 1.7-fold; MGC-803, 2.5-fold; HGC-27, 2.2-fold) compared to GES-1 cells (Fig 1B).

Immunohistochemistry analysis in tumor and adjacent non-tumor normal tissues from a group of gastric cancer patients demonstrated that SIRT3-positive cells were more frequently detectable in tumor tissues than that in normal tissues. The results showed that 55.1% of tumor tissues (7% of normal tissues) exhibited high level (strong positive), 41.4% of tumor tissues (28% of normal tissues) exhibited medium high level (weak positive), while 3.5% of tumor tissues and 64% of normal tissues was negative in SIRT3 expression (Fig 1C and 1D, S1A Fig). Interestingly, SIRT3 expression in intestinal type of tumor tissues (15 cases; 93.3% strong positive and 6.6% weak positive) was significantly higher than that in diffuse type of tumor tissues (14 cases; 14.3% strong positive, 78.6% weak positive and 7.1% negative) (Fig 1C and 1E, S1B Fig). These results suggest that SIRT3 expression is increased in some tumors compared with related normal tissues and SIRT3 expression may be vary in individual tumors, which need to be further investigated.

### SIRT3 levels are linked with cell proliferation

**Fig 2:**
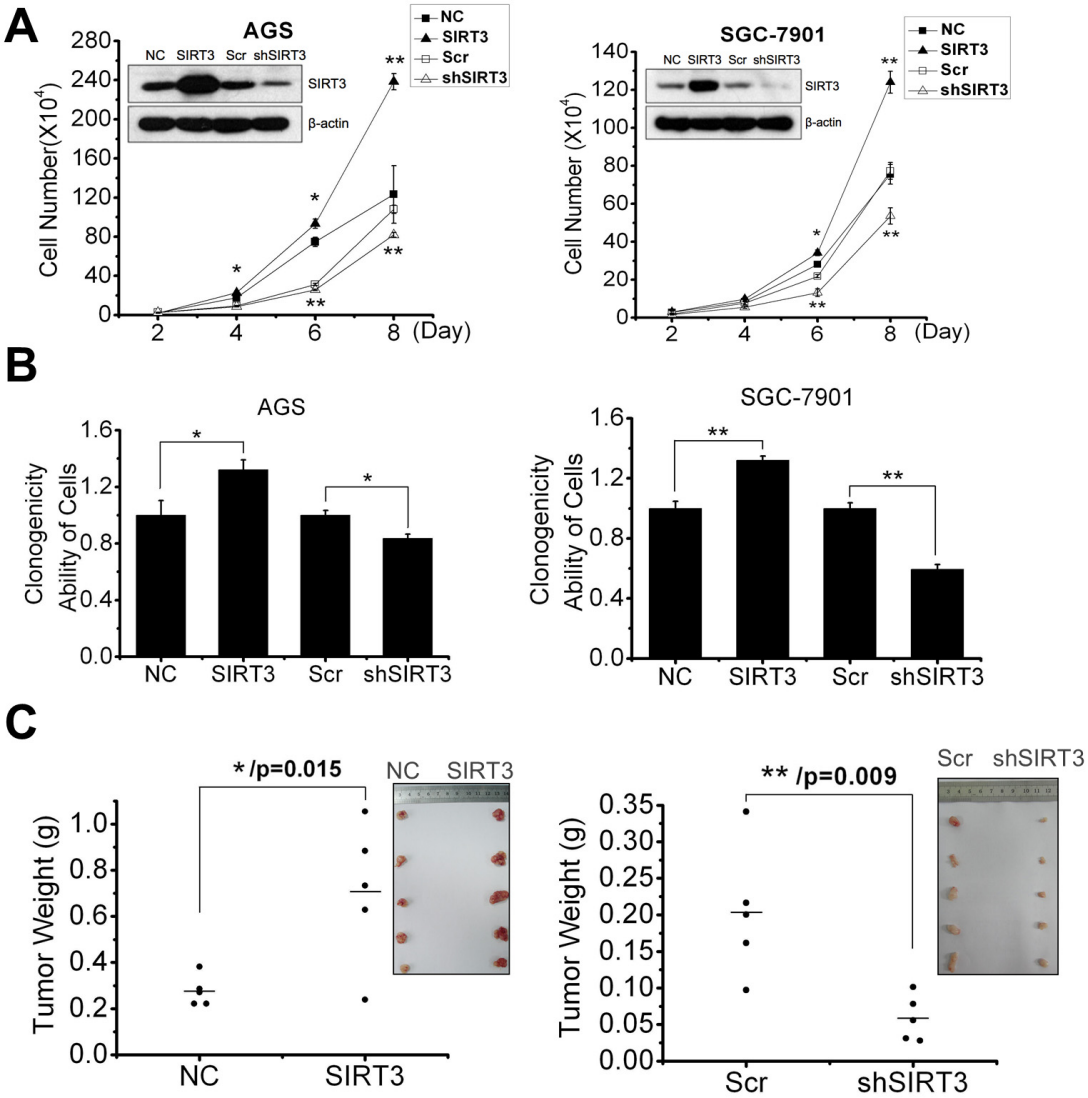
SIRT3 promotes aggressive characteristics of gastric cancer cells. A, cell growth of AGS and SGC-7901 cells with SIRT3 overexpression or knockdown was calculated on days 2, 4, 6 and 8 after cell plating. SIRT3 expression in stable transfectants was confirmed by western blot. B, clonogenicity of AGS and SGC-7901 cells with SIRT3 overexpression or knockdown was measured and presented as the fraction of control transfectants (NC or Scr). C, Overexpression of SIRT3 promoted tumor burden in vivo. SGC-7901 cells with SIRT3 overexpression (left panel) or knockdown (right panel) were subcutaneously injected into right flank of the nude mice with the relative control cells (NC or Scr) into the left flank. Xenograft tumors were excised and weighed at the 28^th^ day after cell inoculation. Images of right panel showed xenograft tumors in vivo at the end of the experiment. Images of up right corner showed the dissected tumors from each group. The ranges and means of tumor weights of each group were presented in right panel as mean ± S.E. (n = 5; *, *p* < 0.05; **, *p* < 0.01). SIRT3, SIRT3 overexpression; shSIRT3, SIRT3 knockdown; NC (negative control; empty pcDNA3.1) and Scr (scramble shRNA; pGPH1/GFP/Neo-shRNA) serve as controls for pcDNA3.1-SIRT3 and pGPH1/GFP/Neo-shSIRT3 respectively.

Then we established SIRT3 overexpression and knockdown cell lines using AGS and SGC-7901 cells. The expression of SIRT3 in stable transfectants was confirmed by western blot. Overexpression of SIRT3 increased cell growth, while SIRT3 knockdown inhibited cell growth of both gastric cancer cell lines (Fig 2A). Further, overexpression of SIRT3 triggered more colony formation than control transfected cells that was in contrast with only fewer colonies formed in the SIRT3 knockdown cells (Fig 2B).

To further determine the role of SIRT3 in tumor formation ability, we injected SIRT3 overexpression and knockdown SGC-7901 cells into flanks of nude mice and tumor growth was allowed for 28 days after cell inoculation (S2 Fig). The average weight of the tumors derived from SIRT3 overexpressing cells was 0.7079g, significantly bigger than the average tumor weight of control (NC), *p* = 0.015, (Fig 2C, left panel). In contrast, the average weight of tumors derived from SIRT3 knockdown cells was 0.0588g, significantly smaller than that from scramble shRNA control cells (Scr) (0.2033g; *p* = 0.009) (Fig 2C, right panel). The average volume of tumors derived from SIRT3 overexpressing cells was increased than that from control cells (NC) (Fig 2C, left panel up right corner). On the contrary, the average volume of tumors growing from SIRT3 knockdown cells was dramatically small compared to that from control cells (Scr) (Fig 2C, right panel up right corner).

### Enhanced glycolysis in SIRT3 expressing gastric cancer cells

**Fig 3:**
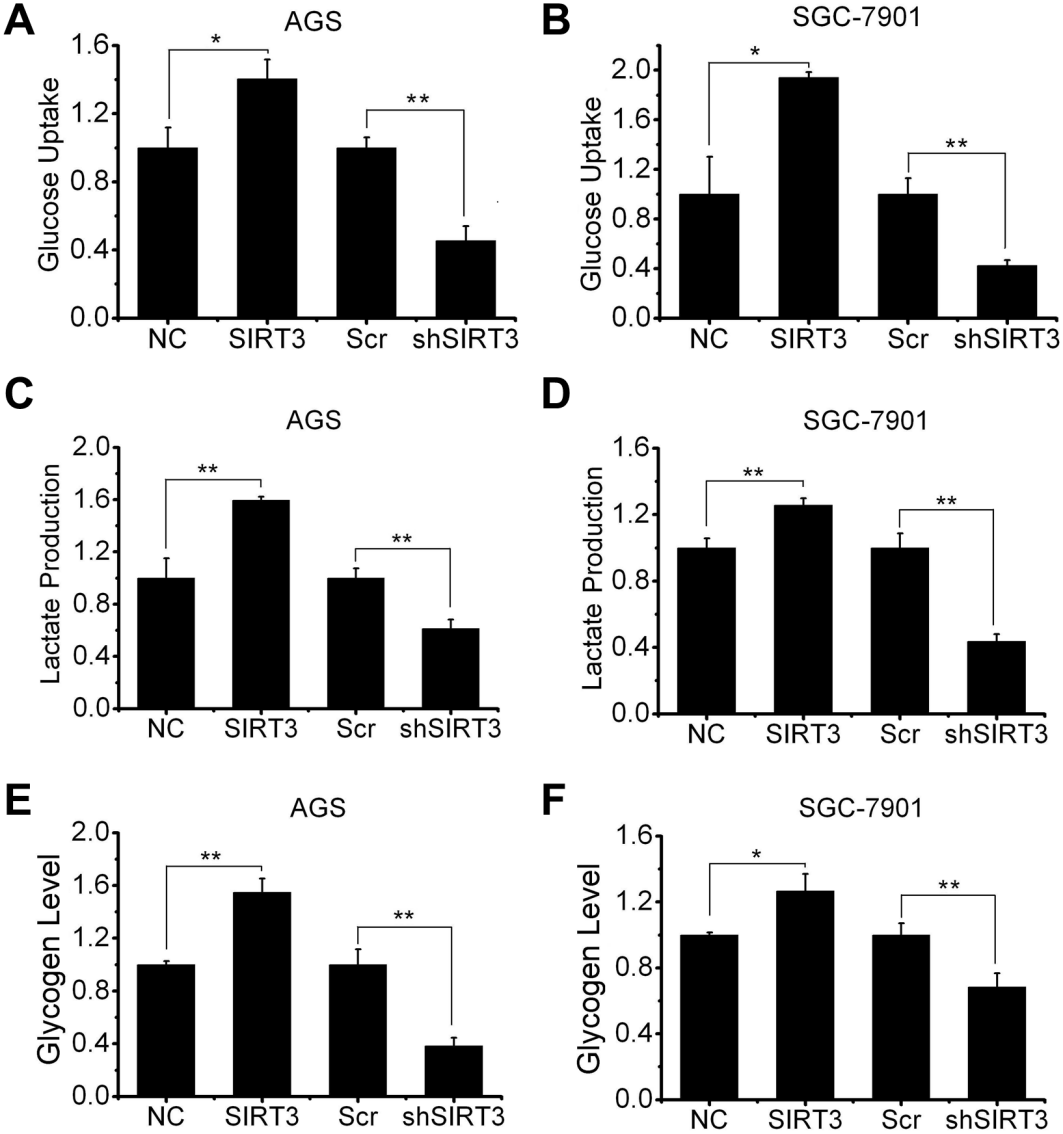
Overexpression of SIRT3 enhances glycolysis in gastric cancer cells. Glucose uptake, lactate production and glycogen formation were measured in AGS (A, C, E) and SGC-7901 (B, D, F) cells with SIRT3 overexpression or knockdown. All data are presented as mean ± S.E. (n = 3; *, *p* < 0.05; **, *p* < 0.01).

We were then wondering how cellular bioenergetics could be altered in SIRT3-overexpressing gastric cancer cells. SIRT3 overexpression dramatically increased glucose uptake (1.4-fold in AGS and 1.9-fold in SGC-7901), while SIRT3 knockdown decreased glucose uptake (around 40% in both AGS and SGC-7901) (Fig 3A and 3B). Consistent with the pattern of glucose usage, SIRT3 overexpressing cells had increased levels of lactate secretion (1.2-fold in AGS and 1.3-fold in SGC-7901), whereas SIRT3 knockdown cells had decreased levels of lactate secretion (55% in AGS and 45% in SGC-7901) (Fig 3C and 3D). We also found that glycogen formation was significantly increased by SIRT3 overexpression (1.5-fold in AGS and 1.3-fold in SGC-7901), a feature of quick growth of tumor cell [33], while reduced by SIRT3 knockdown (40% in AGS and 70% in SGC-7901) (Fig 3E and 3F).

**Fig 4:**
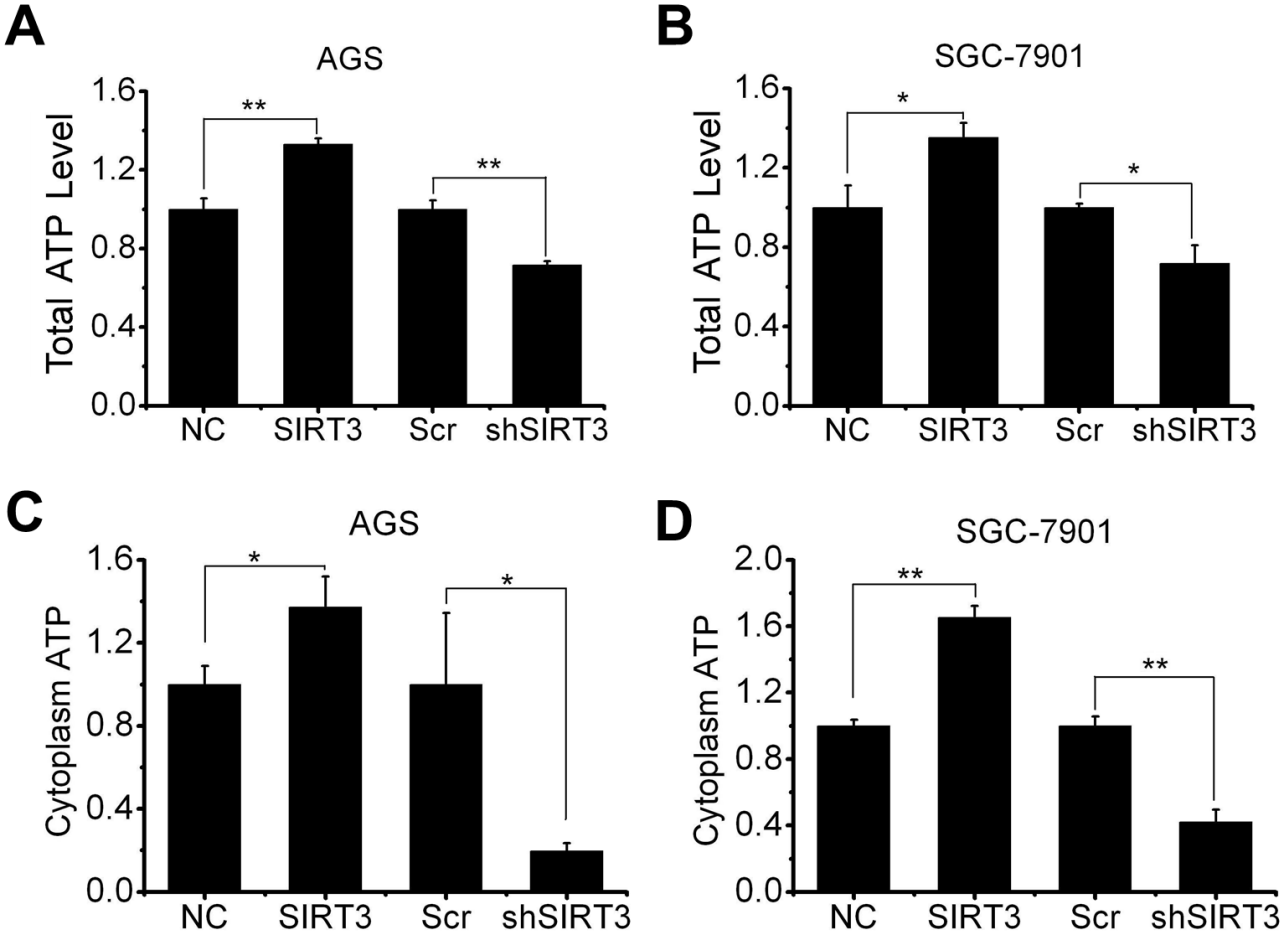
SIRT3 expression is associated with cellular bioenergetics. A and B, total cellular ATP levels were tested in AGS (A) and SGC-7901 (B) cells with SIRT3 overexpression or knockdown. C and D, AGS (C) and SGC-7901 (D) cells with SIRT3 overexpression or knockdown were treated with rotenone (2 μM, 24 hours) to inhibit the oxidative phosphorylation and then cytoplasm ATP levels were measured. Data were normalized by control (NC or Scr) cells and presented as mean ± S.E. (n = 3; *, *p* < 0.05; **, *p* < 0.01).

SIRT3 has been proven involved in the deacetylation of global metabolism enzymes and maintenance of basal ATP levels in cells both in normal condition and in diseases [1,2]. Total cellular ATP and glycolysis ATP generation were detected in AGS and SGC-7901 cells with either SIRT3 overexpression or SIRT3 knockdown. The total cellular ATP levels were increased in SIRT3 overexpressing cells (around 1.3-fold in both cell lines), but decreased in SIRT3 knockdown cells (around 75% in both cell lines) compared to NC or Scr control cells (Fig 4A and 4B). Then we treated cells with rotenone to inhibit oxidative phosphorylation, and tested ATP generation from glycolysis. As is shown in Fig 4C and 4D, SIRT3 overexpression led to an increase in glycolysis ATP production (1.4-fold in AGS and 1.6-fold in SGC-7901), while SIRT3 knockdown led to a significant decrease in glycolysis ATP production (20% in AGS and 40% in SGC-7901) compared to NC or Scr control.

### SIRT3 regulates the homeostasis of ROS in gastric cancer cells

**Fig 5:**
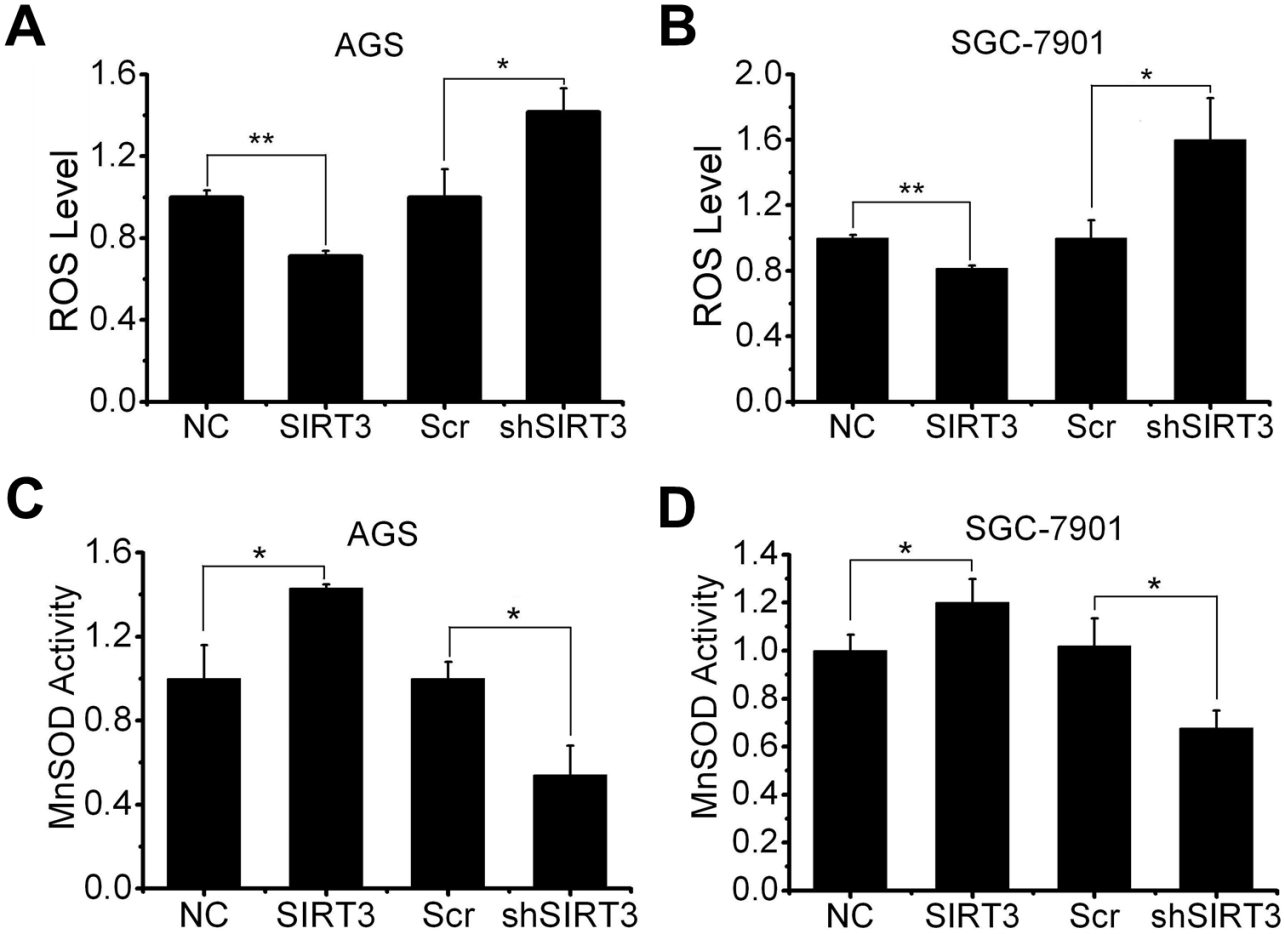
SIRT3 contributes to the regulation of cellular ROS level and MnSOD activity in gastric cancer cells. A and B, cellular ROS levels in AGS (A) and SGC-7901 (B) cells harboring SIRT3 overexpression and knockdown were detected using DCFH2 staining method and represented as relative level normalized by control (NC or Scr). C and D, MnSOD activity was measured in cells mentioned in (A and B) and represented as relative activity normalized by control (NC or Scr). All data are presented as mean ± S.E. (n = 3; *, *p* < 0.05; **, *p* < 0.01).

A body of evidence supports that increased ROS level is a critical characteristic in cancer cells, which make cancer cells more sensitive to additional ROS stress [34]. Here we found that overexpression of SIRT3 decreased ROS levels to 70% and 80% in AGS and SGC-7901 cells, whereas inhibition of SIRT3 expression increased ROS levels (1.4-fold in AGS cells and 1.6-fold in SGC-7901 cells) respectively (Fig 5A and 5B). In agreement with the literature showing SIRT3 deacetylates and activates MnSOD (11, 12), in gastric cancer cells, MnSOD activity was enhanced by SIRT3 overexpression (1.4-fold in AGS cells and 1.2-fold in SGC-7901 cells), but reduced by SIRT3 knockdown (50% in AGS cells and 65% in SGC-7901 cells) (Fig 5C and 5D), suggesting that SIRT3 may protect cells from oxidative stress-induced damage by rebalancing intracellular ROS through enhancing MnSOD activity in gastric cancer cells.

### SIRT3 deacetylates and activates LDHA

**Fig 6:**
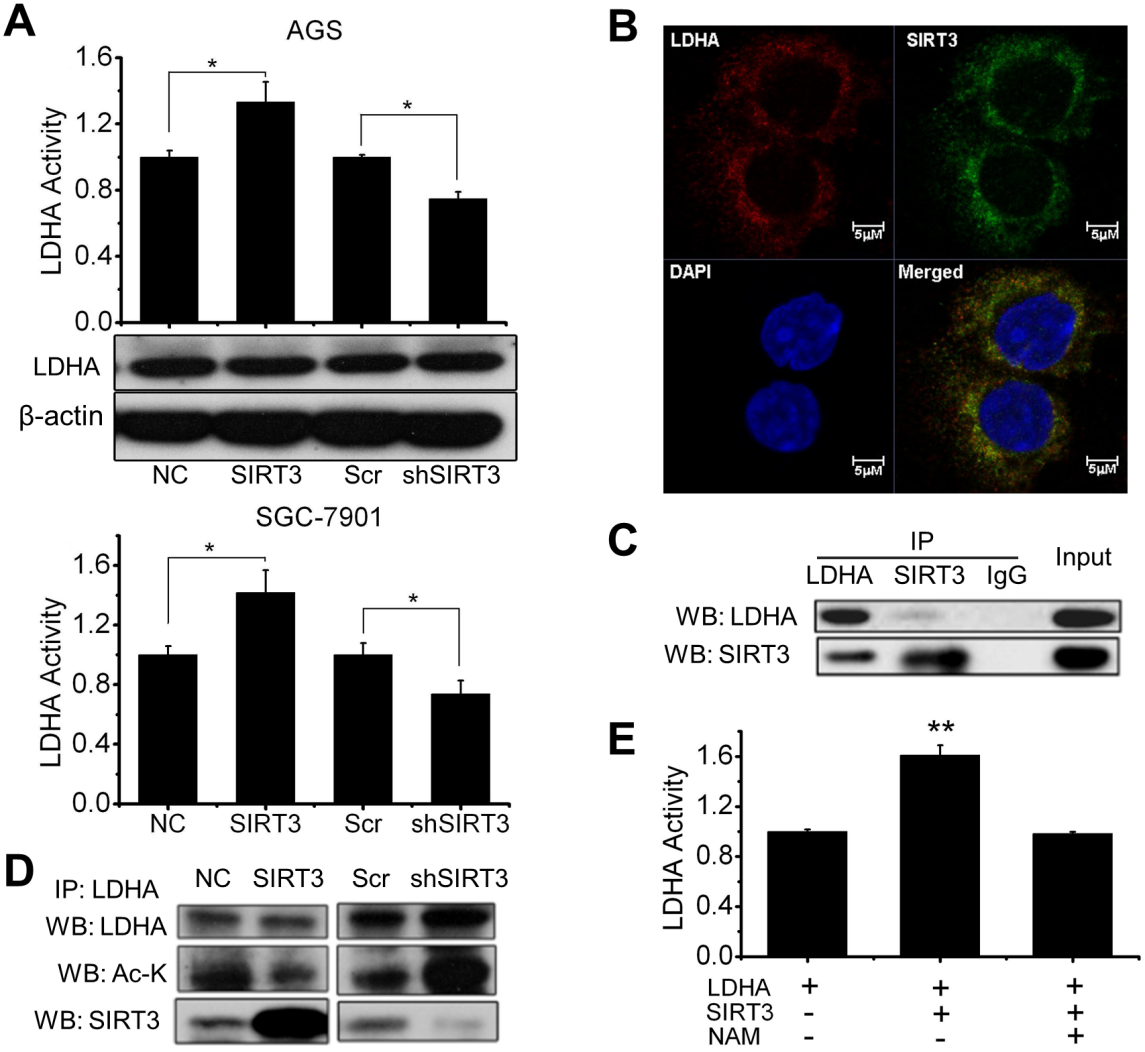
SIRT3 interacts with LDHA and regulates LDHA activity through deacetylation. A, LDHA activity was measured in AGS and SGC-7901 cells with SIRT3 overexpression or knockdown and represented as relative activity normalized by control (NC or Scr). LDHA expression in SIRT3 overexpression and knockdown AGS cells was analyzed by immunoblotting. B, co-localization of SIRT3 and LDHA was detected by immunostaining in AGS cells with anti-SIRT3 (green) and anti-LDHA (red) antibodies. Nuclei were conterstained with DAPI (blue). Scale bar, 5 μm. C, LDHA was immunoprecipitated (IP) followed by western blot (WB) of LDHA or SIRT3, or reverse, in AGS gastric cancer cells. IP with non-immune goat IgG serves as a negative control, and immunoblotting of total cell lysates (input) serves as the IP equal loading control. D, SIRT3-enhanced deacetylation of LDHA in AGS cells was detected by IP with anti-LDHA followed by western blot with anti-LDHA and anti-acetyl-lysine antibodies. Western blot of total cell lysates with SIRT3 antibody to show the expression of transfected protein. E, LDHA enzymatic activity was measured using commercial bovine heart L-Lactic dehydrogenase and recombinant human SIRT3 enzyme with/without SIRT3 inhibitor nicotinamide. F, LDHA enzymatic activity was measured using commercial recombinant human SIRT3 enzyme and immunoprecipitated LDHA from AGS cells transfected with K5Q/R or K318Q/R mutant LDHA with/without SIRT3 inhibitor nicotinamide and presented as relative enzyme activity normalized by wild type LDHA without SIRT3 inhibitor. In A, E and F, data are presented as mean ± S.E. (n = 5; *, *p* < 0.05; **, *p* < 0.01).

LDHA plays a key role in tumor initiation, maintenance and progression. Inhibition of LDHA activity blocks tumorigenicity and tumor progression [26,27] and LDHA activity can be regulated by acetylation/deacetylation modification [35]. We were wondering whether SIRT3 is involved in the regulation of LDHA activity. We found that, in gastric cancer cells, LDHA activity was significantly increased in SIRT3 overexpressing cells, whereas decreased in SIRT3 knockdown cells compared to NC or Scr control cells separately without detectable change in LDHA protein level in the stable transfectants (Fig 6A). Immunostaining results showed that LDHA and SIRT3 were co-localized in the cytoplasm (Fig 6B), and co-IP analysis with either LDHA or SIRT3 antibody revealed that SIRT3 is able to interact with LDHA (Fig 6C). In addition, the level of LDHA acetylation was decreased in SIRT3 overexpressing cells, but increased in SIRT3 knockdown cells (Fig 6D). Further, *in vitro* LDHA activity assay conducted using commercial human recombinant SIRT3 and bovine heart L-Lactic Dehydrogenase indicated that LDHA activity was increased with the presence of SIRT3 in the reaction, which was reversed by the addition of SIRT3 inhibitor, nicotinamide (Fig 6E). To identify potential SIRT3 deacetylation site(s) on LDHA, we searched database and found that K5, K14, K57, K81, K118, K126, K222 and K318 are potential acetylation sites of LDHA, and previous study reported that K5 acetylation/deacetylation was implicated in the regulation of LDHA activity [35]. Then we did LDHA activity assay using lysine 5 and lysine 318 acetylation mimic (K5Q/K318Q) or deacetylation mimic (K5R/K318R) mutant LDHA, and found that neither K5 nor K318 was required for SIRT3-mediated LDHA activation (S3 Fig). Further identification of SIRT3-mediated LDHA deacetylation is in need.

### SIRT3 upregulates genes involved in cellular metabolism

**Fig 7:**
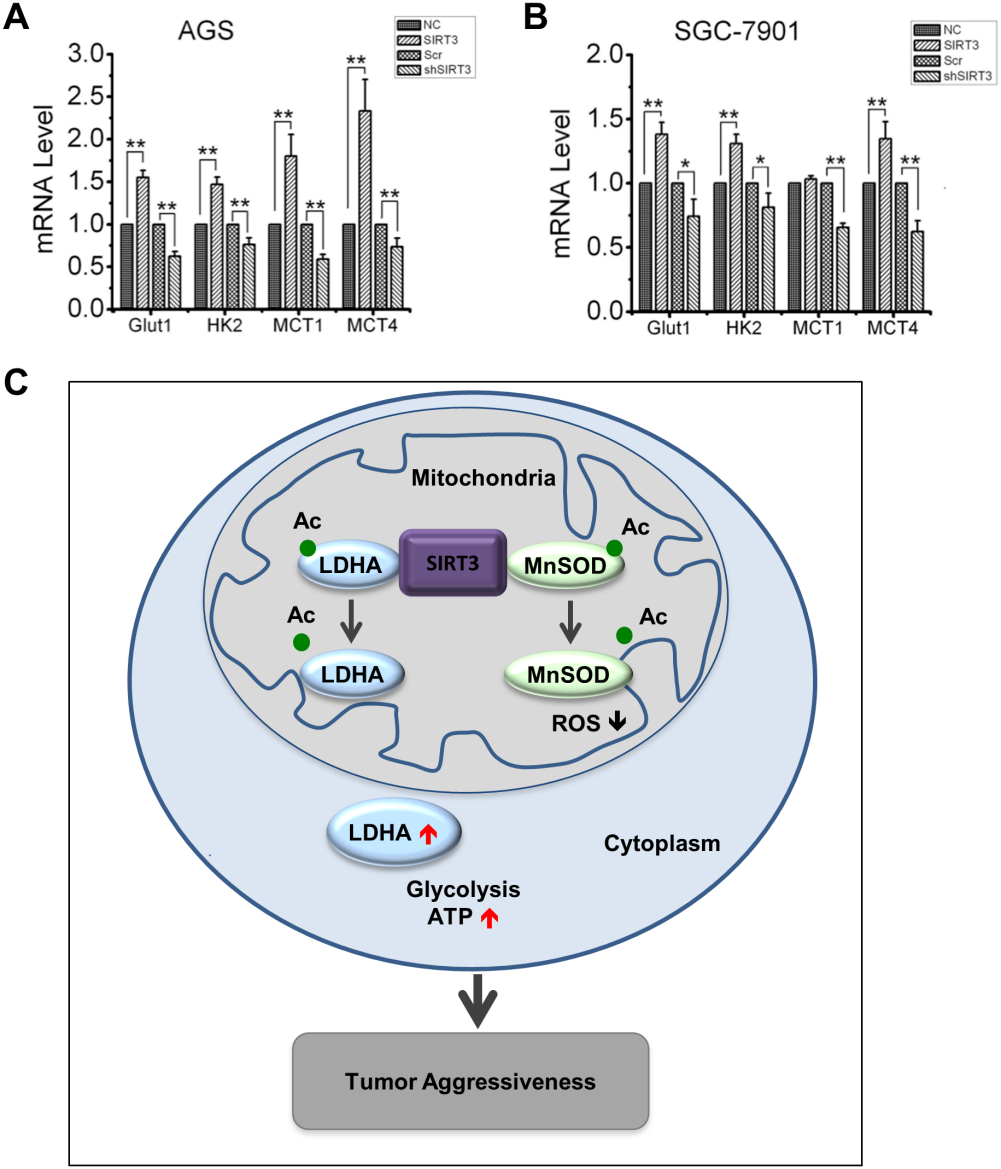
Genes potentially regulated by SIRT3 in gastric cancer cells. Relative mRNA levels of Glut1, HK2, MCT1 and MCT4 were measured by qRT-PCR in AGS (A) and SGC-7901 (B) cells with SIRT3 overexpression or knockdown. Data are presented as mean ± S.E. (n = 3; *, *p* < 0.05; **, *p* < 0.01). (C) Schematic presentation of the potential mechanism by which SIRT3 triggers the cell growth. SIRT3 deacetylates and activates LDHA causing enhanced LDHA activity and cellular bioenergetics, which together with SIRT3-mediated MnSOD activation enhances cell proliferation.

To characterize the molecular signature of SIRT3-LDHA mediated bioenergetics in gastric cancer cells, we measured the expression of genes associated with glucose transportation and glycolysis. Data in Fig 7A and 7B showed that overexpression of SIRT3 in AGS or SGC-7901 induced the expression of HK2 (1.47-fold in AGS cells, 1.3-fold in SGC-7901 cells), MCT4 (2.3-fold in AGS cells, 1.34-fold in SGC-7901 cells) and Glut1 (1.55-fold in AGS cells, 1.38-fold in SGC-7901 cells) at mRNA level tested by real-time PCR. On the contrary, SIRT3 knockdown decreased the expression of gene HK2 to 76%, MCT4 to 73% and Glut1 to 62% in AGS cells, while HK2 to 80%, MCT4 to 62% and Glut1 to 74% in SGC-7901 cells. In addition, when SIRT3 was overexpressed, MCT1 mRNA level was increased in AGS cells by 1.6-fold but not in SGC-7901 cells, and when SIRT3 was knockdown, MCT1 was decreased to 59% in AGS and 65% in SGC-7901 cells, respectively.

## Discussion

This study provides the evidence indicating that SIRT3 expressed in some cancer cells, such as gastric cancer cells, may be linked with the enhanced aggressiveness by SIRT3-mediated bioenergetics via deacetylation and activation of LADH. Although accumulating evidences indicates that SIRT3 plays a key role in protection of normal cells against aging and malignant transformation, its function in already transformed cells such as tumor cells that express SIRT3 needs to be investigated [19]. Lack of SIRT3 in MEFs leads to genomic instability and a high frequency of cell transformation mediated by Ras with an increased rate of tumor formation and aging, suggesting that SIRT3 is necessary in prevention of cellular aging and carcinogenesis [11,15]. In addition, lack of or lower expression of SIRT3 is detected in several human cancers and SIRT3 level is associated with the sensitivity of cancer cells to chemo- and radiotherapy [11,15,16]. However, SIRT3 is also shown be overexpressed in human cancers and associated with therapy resistance [19,21,36]. These results suggest that SIRT3 may target different proteins between normal and cancer cells, and that SIRT3 expression can be varied in different types of cancer, such as the current results indicating that the intestinal type of gastric cancer expresses a higher level of SIRT3 than the diffuse type of gastric cancer.

It has been generally accepted that tumor cells consume more cellular energy to support the fast growth and proliferation than the normal cells using glycolysis as the main source of ATP generation instead of oxidative phosphorylation [24]. In current study, we demonstrated that, in gastric cancer cells, SIRT3 interacts with and deacetylates LDHA, causing increased LDHA activity; and SIRT3 overexpression leads to increased cellular ATP production and MnSOD activity paralleled with reduced cellular ROS level, indicating an increased oxidative scavenger ability of SIRT3 possibly via deacetylation-mediated MnSOD enzyme activation. Increasing evidence shows that SIRT3 is required for the maintenance of cellular and mitochondria homeostasis through regulating mitochondria metabolism and cellular redox balance system, protecting cells against oxidative stress via regulating ROS generation, a critical mechanism in aging, cancer and heart diseases [1,2,37]. SIRT3 can deacetylate a group of mitochondria targets implicated in the regulation of both glycolysis and cellular oxidative stress. Recently, SIRT3 is found be able to deacetylate and increase pyruvate dehydrogenase activity in cancer cells, which can increase both mitochondrial bioenergetics and glycolysis [38]. In agreement, the current study indicates that SIRT3 can deacetylate and activate LDHA that can be used for both mitochondrial respiration and glycolysis. On the other hand, SIRT3 can deacetylate and subsequently activate other mitochondria substrates, such as MnSOD [11,12], Foxo3 [39] and IDH2 [9] to scavenge ROS produced by oxidative phosphorylation, protecting cells from the oxidative damage [40]. Also consistent with these findings, in this study, we found that overexpression of SIRT3 increases MnSOD activity, which could be one reason of reduced cellular ROS level under enhanced cellular metabolism condition.

LDHA, as a family member of lactate dehydrogenase (LDH), is found existed in glycolytic cells and localized in mitochondria in addition to the cytosol [41], and mainly catalyzing the inter-conversion of pyruvate and lactate [42]. LDHA is shown to play a key role in aerobic glycolysis and tumorigenicity in malignant cells [26,27]. In cancer cells, LDHA rapidly consumes pyruvate produced by glycolytic pathway, leading to increased aerobic lactate production [26]. Inhibition of LDHA induces ROS production, decreases cellular ATP levels and inhibits glycolysis, therefore inhibits cell growth and triggers cell death in cancer [27]. Consisting with these reports, we found that SIRT3 can interact and activate LDHA. In the SIRT3 overexpressing cells, LDHA acetylation was decreased with an increased LDHA enzyme activity. Although exact mechanism causing SIRT3-mediated LDHA regulation needs to be further investigated, these results demonstrate that LDHA controlled glycolysis pathway that accelerates tumor bioenergetics can be affected by SIRT3 expression levels.

Our current study also revealed that the expression of a group of genes, such as MCT1, MCT4, HK2 and Glut1, known as key regulators of energy metabolism in cancer are also increased in SIRT3-overexpressing gastric cancer cells, demonstrating a potential role of SIRT3 in enhancing glycolysis in tumor cells. HK2 catalyzes phosphorylation of glucose to glucose-6-phosphate at the initial step of glycolysis [43]. Glucose transporter (GLUT) controls the transport of glucose across the plasma membrane, functioning as the first rate-limiting step for glucose metabolism [44]. MCT1 and MCT4, members of monocarboxylates (such as lactate and pyruvate) transporter family, are also involved in the growth of glycolysis-dependent tumors [45]. Together, these results suggest a potential interplay between SIRT3 and a group of glycolysis-associated genes in signaling tumor aggressive growth, which needs to be further investigated.

In conclusion, although SIRT3 is well characterized in mitochondrial homeostasis against cell aging and transformation, expression of SIRT3 in tumor cells is linked with enhanced proliferation and aggressive growth of gastric cancer cells. SIRT3 interacts and activates LDHA, leading to increased glycolysis and ATP production, which together with increased mitochondrial antioxidant MnSOD activity and decreased intracellular ROS level (a hypothetic model is proposed in Fig 7C). Thus, the growth-stimulating function of SIRT3 demonstrates a potential target to treat tumors with SIRT3 expression.

## Supporting Information

S1 Fig Representative pathological slides for determining the SIRT3 expression in human gastric cancer specimens. A, paraffin-embedded human gastric cancer and paired adjacent pathologically normal tissue specimens were subjected to immunohistochemistry staining with SIRT3 antibody (brown) followed by nuclei counterstain with hematoxylin (blue; each tumor was shown with adjacent normal tissues in one row by 20× and 40×). B, representative images of SIRT3 expression in the intestinal and diffuse types of gastric cancer. Scale bar, 50 μm.
(TIF)

S2 Fig Overexpression of SIRT3 promoted tumor burden in vivo. SGC-7901 cells with SIRT3 overexpression (A) or knockdown (B) were subcutaneously injected into right flank of the nude mice with the relative control cells (NC or Scr) into the left flank. Xenograft tumors were excised and weighed at the 28th day after cell inoculation. Images of right panel showed xenograft tumors in vivo at the end of the experiment. Images showed tumor growth in mice.
(TIF)

S3 Fig K5 and K318 of LDHA are not specific deacetylation sites of SIRT3. LDHA enzymatic activity was measured using commercial recombinant human SIRT3 enzyme and immunoprecipitated LDHA from AGS cells transfected with K5Q/R (A) or K318Q/R (B) mutant LDHA with/without SIRT3 inhibitor nicotinamide and presented as relative enzyme activity normalized by wild type LDHA without SIRT3 inhibitor. Data are presented as mean ± S.E. (n = 5; *, p < 0.05; **, p < 0.01).
(TIF)

S1 Table Primer sequence for real-time PCR. (XLSX)
