# Supplementary figures and images for: SIRT3 Enhances Glycolysis and Proliferation in SIRT3-Expressing Gastric Cancer Cells
Source: PLoS One. 2015 Jun 29;10(6):e0129834. doi: 10.1371/journal.pone.0129834 (PMC4487898; doi:10.1371/journal.pone.0129834)

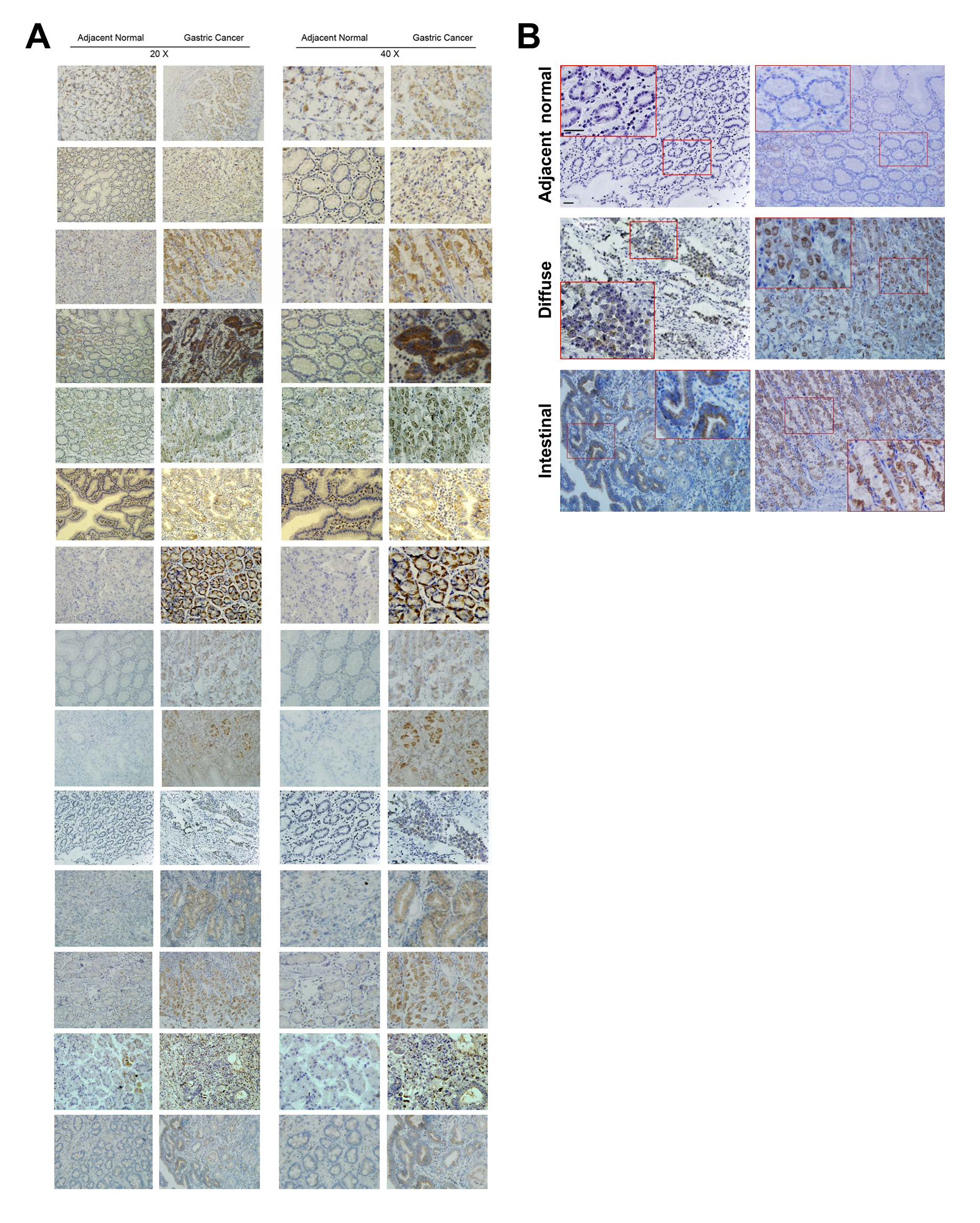

Supplement: S1 Fig — A, paraffin-embedded human gastric cancer and paired adjacent pathologically normal tissue specimens were subjected to immunohistochemistry staining with SIRT3 antibody (brown) followed by nuclei counterstain with hematoxylin (blue; each tumor was shown with adjacent normal tissues in one row by 20× and 40×). B, representative images of SIRT3 expression in the intestinal and diffuse types of gastric cancer. Scale bar, 50 μm. (TIF) [file pone.0129834.s001.tif]

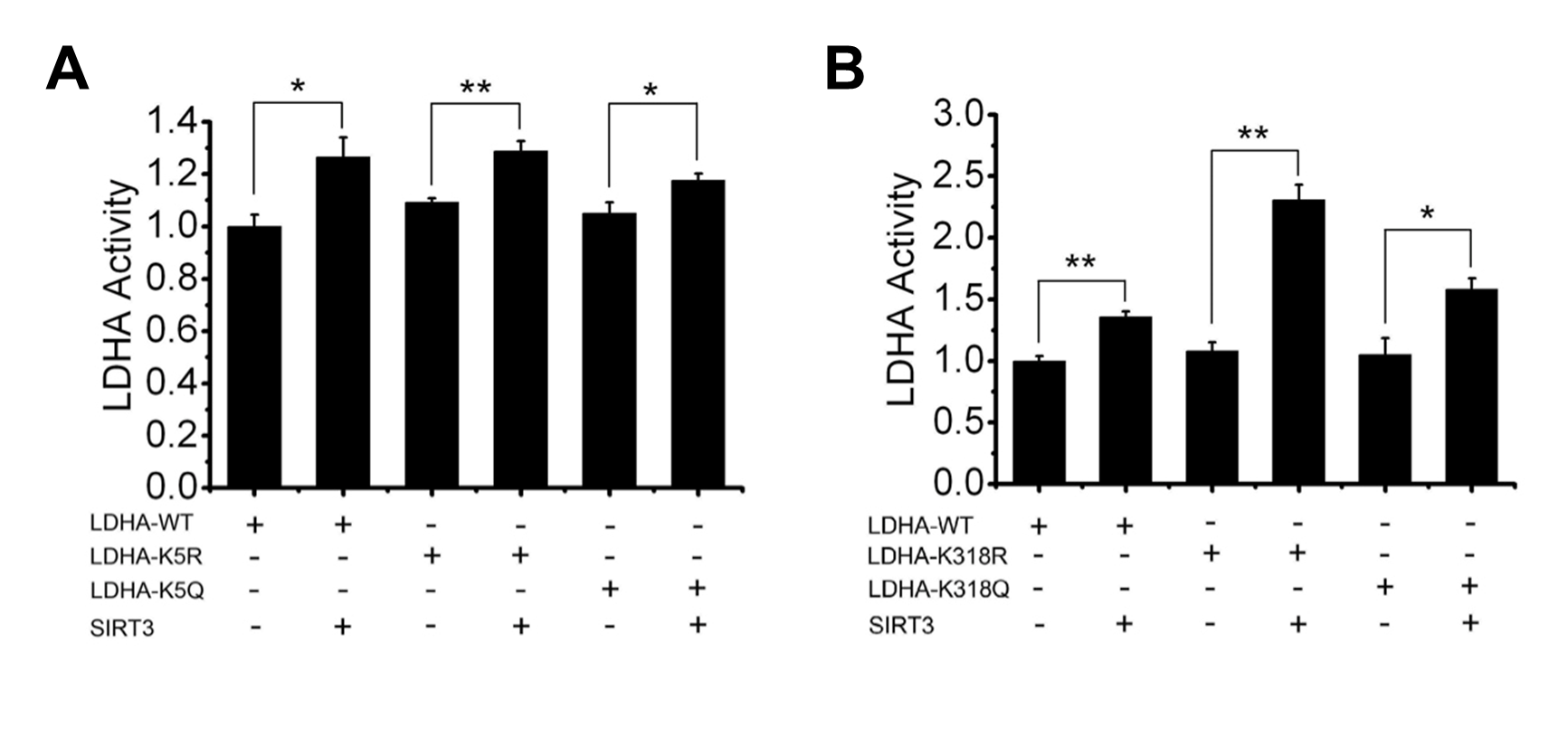

Supplement: S2 Fig — SGC-7901 cells with SIRT3 overexpression (A) or knockdown (B) were subcutaneously injected into right flank of the nude mice with the relative control cells (NC or Scr) into the left flank. Xenograft tumors were excised and weighed at the 28th day after cell inoculation. Images of right panel showed xenograft tumors in vivo at the end of the experiment. Images showed tumor growth in mice. (TIF) [file pone.0129834.s002.tif]

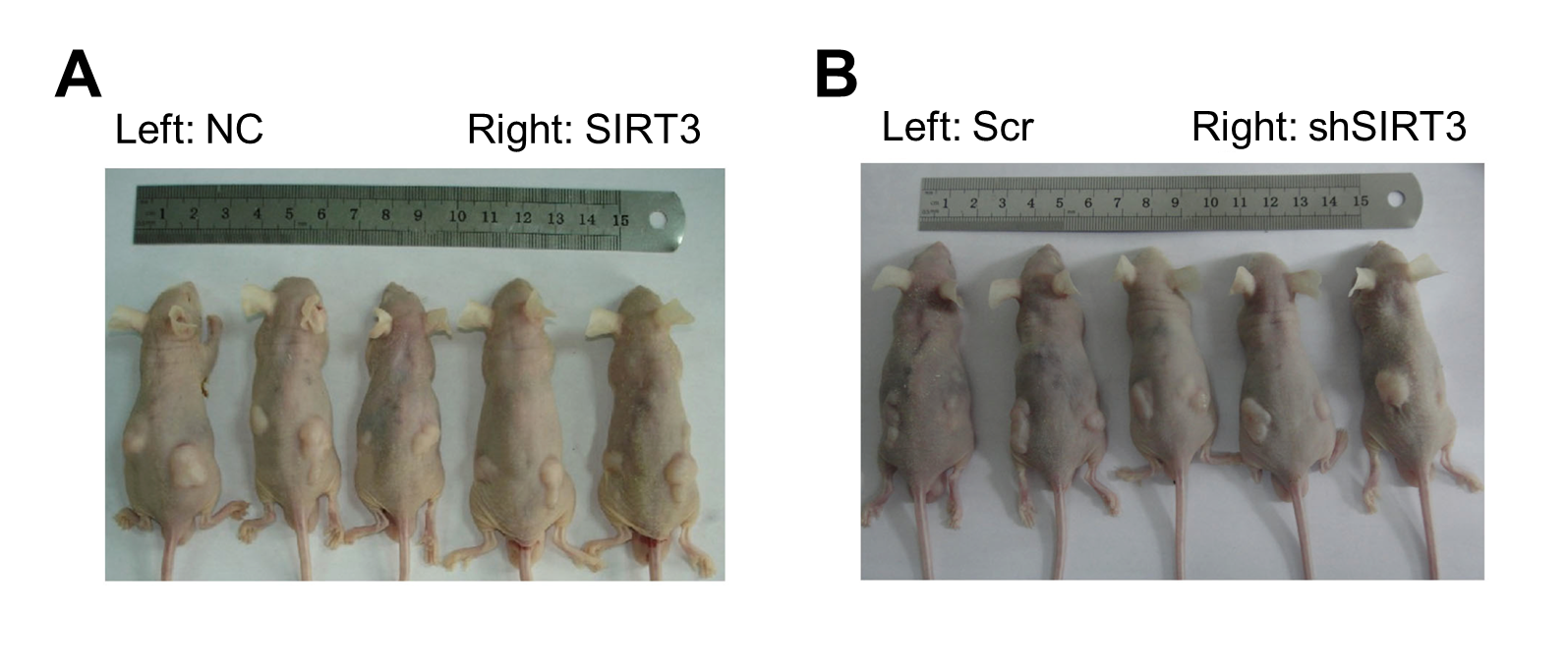

Supplement: S3 Fig — LDHA enzymatic activity was measured using commercial recombinant human SIRT3 enzyme and immunoprecipitated LDHA from AGS cells transfected with K5Q/R (A) or K318Q/R (B) mutant LDHA with/without SIRT3 inhibitor nicotinamide and presented as relative enzyme activity normalized by wild type LDHA without SIRT3 inhibitor. Data are presented as mean ± S.E. (n = 5; *, p < 0.05; **, p < 0.01). (TIF) [file pone.0129834.s003.tif]
